# Retracting PetAls againST the sEptum (Re-PASTE) technique for right inferior pulmonary vein pentaspline pulsed field ablation: A case report

**DOI:** 10.1016/j.ipej.2025.10.005

**Published:** 2025-10-15

**Authors:** Yuhei Kasai, Takayuki Kitai, Junji Morita, Kazuhiro Satomi

**Affiliations:** a Department of Cardiology, Sapporo Cardiovascular Clinic, Sapporo, Hokkaido, Japan; b Department of Cardiology, Tokyo Medical University, Tokyo, Japan

**Keywords:** Pulmonary vein isolation, Pulsed field ablation, Flower configuration, Petals, Radiopaque marker

## Abstract

Pulsed field ablation is a nonthermal ablation modality with favorable safety and efficacy profiles for pulmonary vein isolation in patients with atrial fibrillation. However, isolating the right inferior pulmonary vein can be technically challenging because of anatomical limitations and restricted catheter maneuverability. We report the case of a 47-year-old man with symptomatic, drug-refractory paroxysmal atrial fibrillation who underwent successful pulsed field ablation under general anesthesia using the FARAPULSE system. The Retracting the PetAls (in the flower configuration) againST the sEptum (Re-PASTE) technique was used in combination with radiopaque markers on the pentaspline FARAWAVE catheter and FARADRIVE sheath to facilitate accurate right inferior pulmonary vein access using fluoroscopic guidance alone. The Re-PASTE technique benefits from the design features of the pentaspline PFA catheter and sheath, such as their widths and lengths and their radiopaque markers, thereby enabling clear identification of the right–left atrial boundary and stable catheter positioning without spline deformation. The technique is simple, reproducible, and effective in addressing right inferior pulmonary vein anatomical challenges. The Re-PASTE technique may enhance procedural safety and efficiency, particularly in cases with difficult right inferior pulmonary vein anatomy, offering a practical solution to a common technical limitation in pulsed field ablation procedures.

## 1 Introduction

Pulsed field ablation (PFA) is a novel nonthermal modality for atrial fibrillation (AF) ablation, with well-established efficacy and safety profiles [1]. Unlike thermal energy sources, such as radiofrequency and cryoballoon ablation, PFA is associated with a lower incidence of collateral tissue complications [2]. Among different kinds of PFA catheters, real-world data have shown the procedural effectiveness of the pentaspline PFA catheter (FARAWAVE™, Boston Scientific, Marlborough, MA, USA) for pulmonary vein isolation (PVI) [3]. However, isolating the right inferior pulmonary vein (RIPV) can be technically challenging with the pentaspline catheter, particularly when the Brockenbrough puncture site is positioned near the RIPV. In such cases, limited maneuvering space may lead to spline bunching, impeding manipulation of the catheter. Severe bunching can result in a “cobra-like” deformation, often necessitating catheter withdrawal, increasing the risk of air embolism [4]. To address this challenge, we report a case in which the Retracting the PetAls (in the flower configuration) againST the sEptum (Re-PASTE) technique allowed clear identification of the right–left atrial boundary, while the radiopaque markers on the FARAWAVE catheter and FARADRIVE sheath were used to facilitate precise selection of the RIPV.

## 2 Case report

**Fig. 1:**
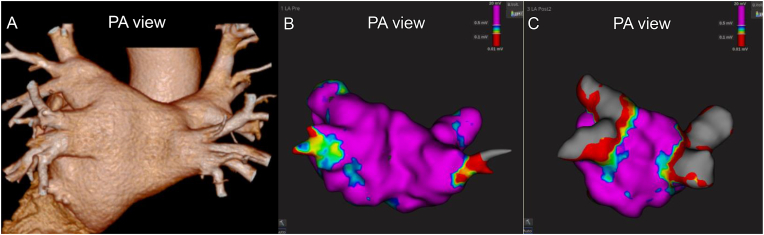
CT and three-dimensional mapping of the left atrium. **(A)** Pre-procedural three-dimensional CT of the pulmonary vein. **(B)** Pre-mapping using the Rhythmia system (Boston Scientific). **(C)** Post-mapping confirmed bilateral PVI after four PFA applications in the basket configuration and four in the flower configuration for each vein. PA, posterior–anterior; CT, computed tomography; PVI, pulmonary vein isolation; PFA, pulsed field ablation.

**Patient's Information.** A 47-year-old man with symptomatic drug-refractory paroxysmal AF underwent PFA under general anesthesia (Fig. 1A).

**Preparation.** After obtaining right femoral venous access and performing transseptal puncture under intracardiac echocardiography guidance, we created a left atrial electroanatomical map using a high-density mapping catheter (ORION, Boston Scientific) (Fig. 1B).

**Left pulmonary vein isolation.** We introduced a steerable sheath (FARADRIVE) into the left atrium. Ablation of the left superior and inferior pulmonary veins was performed using four PFA applications in the basket configuration and four PFA applications in the flower configuration for each vein.

**Right inferior pulmonary vein isolation (our proposed method).**

**Fig. 2:**
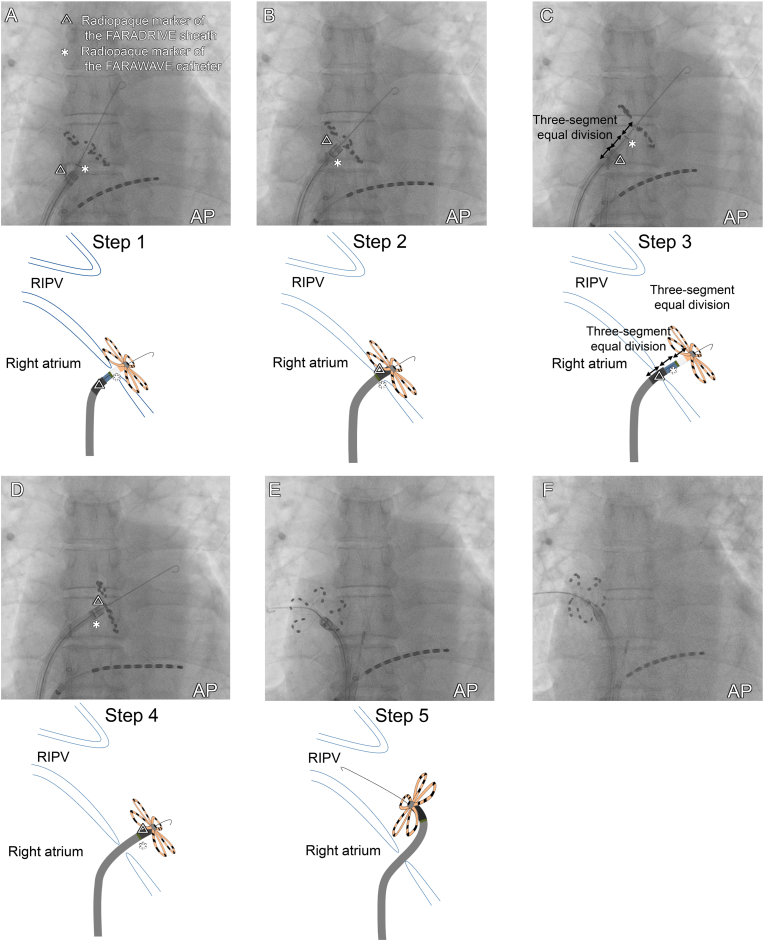
Fluoroscopic images (top) and schemas of the catheter movements (bottom) for completing left PVI to select the RIPV. **(A)** After left PVI, the FARAWAVE catheter and the FARADRIVE sheath were retracted, while the FARAWAVE catheter, in its flower configuration, was pressed against the septum to confirm the right–left atrial boundary. **(B)** The FARADRIVE sheath was advanced as close as possible to the flower configuration while ensuring that the FARAWAVE catheter remained stable. **(C)** The FARAWAVE catheter was advanced until the catheter's radiopaque marker was positioned between the sheath tip and the flower configuration, while maintaining the position of the FARADRIVE sheath. The sheath's radiopaque marker length and the catheter's marker-to-flower distance measuring 8 mm ensured that advancing the catheter exactly 8 mm beyond the sheath tip could be precisely guided under fluoroscopic observation. This approach was based on the three-segment equal division principle. **(D)** The FARADRIVE sheath was advanced in the same manner as that described in (B). With the catheter stationary, the sheath is advanced until its tip protrudes 16 mm into the left atrium. **(E)** The guidewire was advanced into the RIPV, while the FARADRIVE sheath was rotated clockwise. Due to the 15-mm long axis of each petal, careful manipulation of the sheath allows atrial wall contact without causing spline deformation. **(F)** The FARAWAVE catheter was pressed against the RIPV, and PFA applications were performed. AP, anterior–posterior; RIPV, right inferior pulmonary vein.
Step 2 : the FARADRIVE sheath was advanced as close as possible to the flower configuration while maintaining the position of the FARAWAVE catheter (Fig. 2B). Step 3: the FARAWAVE catheter was then advanced until its radiopaque marker was positioned between the sheath tip and the flower configuration, ensuring that the FARADRIVE sheath remained in place. The sheath's radiopaque marker length and the catheter's marker-to-flower distance each measured 8 mm. This approach ensured that advancing the catheter's radiopaque marker exactly 8 mm beyond the sheath tip could be accurately guided using the three-segment equal division method under fluoroscopic guidance, thereby enabling precise positioning (Fig. 2C).
Step 4 : the FARADRIVE sheath was then advanced again as close as possible to the flower configuration (Fig. 2D).
Step 5 : a guidewire was successfully introduced into the RIPV while the FARADRIVE sheath was rotated clockwise (Fig. 2E; Supplementary Video 2). Finally, the FARAWAVE catheter was positioned against the RIPV, and four PFA applications were performed in the flower configuration (Fig. 2F), followed by four PFA applications in the basket configuration.

Step 1 : after the completion of left PVI, the FARAWAVE catheter, in its flower configuration, was retracted and pressed against the septum to confirm the right–left atrial boundary (Fig. 2A; Supplementary Video 1).

**Right inferior pulmonary vein isolation and post-map.** Subsequently, in the right superior pulmonary vein, four PFA applications were delivered in the basket configuration, followed by four applications in the flower configuration. Post-mapping with a high-density mapping catheter confirmed bilateral PVI (Fig. 1C).

**Results.** The procedure, including all of the five steps, was finished without complications. The patient has remained free of AF recurrence during a 12-month follow-up.

## 3 Discussion

PFA is a non-thermal technology using electric fields for selective myocardial cell destruction, reducing collateral damage and complications [1]. Previous reports have shown that the RIPV presents greater challenges than other pulmonary veins because of anatomical difficulties and limited maneuverability [4,5]. One potential solution is to retract the PFA catheter into the FARADRIVE sheath and advance a guidewire alone across the RIPV, but this approach carries the risk of the sheath slipping back into the right atrium, especially when the RIPV has a more inferiorly positioned ostium. Our Re-PASTE technique enables clear identification of the right–left atrial boundary and stable catheter positioning, thereby avoiding such risks.

**Fig. 3:**
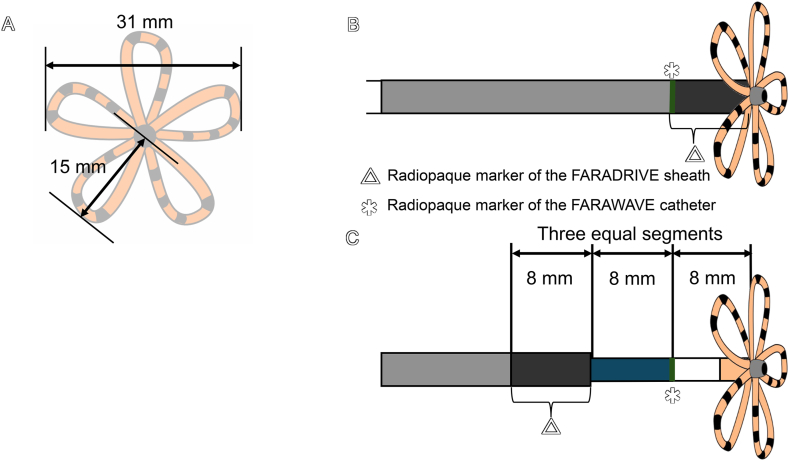
Structural components of the FARAWAVE catheter and FARADRIVE sheath with radiopaque markers. **(A)** The FARAWAVE catheter has a 31-mm opposing petal distance in the flower configuration and the FARADRIVE sheath measures 16.8 Fr (5.6 mm). Therefore, the FARAWAVE catheter remains within the left atrium even when retracted in the flower configuration, preventing inadvertent passage into the right atrium (see Fig. 2, Fig. 3A). **(B)** The sheath's radiopaque marker length and the catheter's marker-to-flower distance both measure 8 mm. Therefore, advancing the FARADRIVE sheath as close as possible to the flower configuration ensures precise alignment of the catheter's radiopaque marker with the proximal edge of the sheath's radiopaque marker, (see Fig. 2, Fig. 3D). **(C)** Advancement of the catheter's radiopaque marker 8 mm beyond the sheath tip while maintaining the sheath at the right-left atrial boundary is facilitated by fluoroscopic guidance, effectively dividing the 8-mm distance into three equal segments and ensuring precise positioning (see Fig. 2, Fig. 3C).

All five steps of Re-PASTE take advantage of the characteristics of the FARAWAVE catheter, FARADRIVE sheath, and their radiopaque markers, suggesting that the technique can be applied to a wide range of cases. In Step 1 (see the case report section, Fig. 2A), the width gap between the sheath and petals enables clear delineation of the right–left atrial boundary. The FARAWAVE catheter has an opposing petal distance of 31 mm in the flower configuration and the FARADRIVE sheath measures 16.8 Fr (5.6 mm). Therefore, retracting both devices still lead to anterior petal folding, making inadvertent dislodgement into the right atrium unlikely because of the size of the iatrogenic atrial septal defect (Fig. 3A).

In Steps 2 and 4, establishing the precise orientation under fluoroscopic guidance using radiopaque markers of the FARAWAVE and FARADRIVE catheters is crucial. Advancing the FARADRIVE sheath as close as possible to the flower configuration ensures precise alignment of the catheter's radiopaque marker with the proximal edge of the sheath's radiopaque marker, thereby ensuring accurate and reproducible catheter positioning (Fig. 2, Fig. 3B).

Steps 3 and 5 leverage three equal 8-mm segments, making catheter positioning highly reproducible during ablation. By advancing the FARAWAVE catheter and FARADRIVE sheath 16 mm into the atrial boundary, operators gain space for deflection and rotation without spline deformation (each petal is 15 mm long, Fig. 3A). Fluoroscopy then confirms the catheter marker 8 mm beyond the sheath tip (Fig. 2, Fig. 3C), ensuring precise placement. This case highlights a practical RIPV selection technique under fluoroscopic guidance alone, emphasizing the importance of understanding the characteristics of the catheter and sheath.

## 4 Conclusion

The Re-PASTE technique provides a straightforward and reproducible method for precise RIPV access by benefiting from the characteristics of pentaspline PFA catheters and sheaths. By relying solely on fluoroscopic visualization of radiopaque markers, this approach enables clear delineation of the right–left atrial boundary and stable catheter positioning without deformation. The simplicity and effectiveness of this technique make it a useful option for overcoming common anatomical challenges in PFA procedures, and it may contribute to improved procedural safety and efficiency.

## Patient's consent

Written informed consent was obtained from the patient for the publication of this case report and any accompanying images.

## Ethical statement

All procedures were performed in compliance with relevant laws and institutional guidelines and have been approved by the appropriate institutional committee. The privacy rights of human subjects have been observed. The authors confirm that written consent for submission of this report was obtained from the patient, in accordance with the COPE guidelines.

## Author contributions

Yuhei Kasai: Writing – original draft, Methodology.

Takayuki Kitai and Junji Morita: Supervision, Writing – review and editing.

Kazuhiro Satomi: Supervision, Project administration.

## Ethical statement

All procedures were performed in compliance with relevant laws and institutional guidelines and have been approved by the appropriate institutional committee. The privacy rights of human subjects have been observed. The authors confirm that written consent for submission of this report was obtained from the patient, in accordance with the COPE guidelines.

## Funding

This research did not receive any specific grant from funding agencies in the public, commercial, or not-for-profit sectors.

## Declaration of competing interest

The authors declare that they have no known competing financial interests or personal relationships that could have appeared to influence the work reported in this paper.

## Appendix A Supplementary data

Supplementary Video 1 This video demonstrates the FARAWAVE catheter in its flower configuration being gently retracted and pressed to the atrial septum, with the petals subtly tilting in a distal direction to delineate the right–left atrial boundary under fluoroscopic guidance Supplementary Video 1

Supplementary Video 2 With the FARADRIVE sheath theoretically advanced such that its tip protruded 16 mm into the left atrium, the sheath was rotated clockwise, allowing smooth advancement of the guidewire into the RIPV Supplementary Video 2

## References

[1] Reddy V.Y., Neuzil P., Koruth J.S. (2019). Pulsed field ablation for pulmonary vein isolation in atrial fibrillation. J Am Coll Cardiol.

[2] Ekanem E., Neuzil P., Reichlin T. (2024). Safety of pulsed field ablation in more than 17,000 patients with atrial fibrillation in the MANIFEST-17K study. Nat Med.

[3] Kueffer T., Bordignon S., Neven K. (2024). Durability of pulmonary vein isolation using pulsed-field ablation: results from the multicenter EU-PORIA registry. JACC Clin Electrophysiol.

[4] Ekanem E., Neuzil P., Reichlin T. (2024). Safety of pulsed field ablation in more than 17,000 patients with atrial fibrillation in the MANIFEST-17K study. Nat Med.

[5] Badertscher P., Knecht S., Rosso R. (2025). How to perform pulmonary vein isolation using a pentaspline pulsed field ablation system for treatment of atrial fibrillation. Heart Rhythm.

